# 

**Published:** 1868-07

**Authors:** 


### TO THE AMERICAN DENTAL ASSOCIATION.

As an inducement to many of our professional brethren, of this part of the country, to be present at the meeting of this Association, on the 28th inst., we are happy to announce the following arrangement.

ROUND-TRIP TICKETS,

Will be sold by the Cincinnati, Hamilton & Dayton Railroad, from Cincinnati to Niagara Falls and return, for $14 50. Passengers with these tickets have the privilege of stopping off at Toledo and visiting Put-in-Bay and islands in Lake Erie. The splendid side-wheel steamer "Lake Breeze" leaves Toledo every morning at 8 o'clock for Kelley's Island, Gibraltar and Put-in-Bay, returning the same evening.

Two Express Passenger Trains leave Cincinnati daily, as follows: Morning Express, 6:15, arriving at Toledo 3:50 P. M., Detroit 6:00 P. M., Niagara Falls 4:00 A. M. Night Express, 7:10 P. M., arriving at Toledo 5:45 A. M., Detroit at 8:30 A. M., and Niagara Falls at 3:45 P. M.

These trains run through from Cincinnati to Niagara Falls with only one change of cars. In order to reach the falls in time for the meeting it will be necessary to take the 6:15 A. M. train at C. H. & D. Depot, on the 27th inst., which will arrive at Niagara next morning at 4 o'clock. Persons may take advantage of this arrangement by taking the cars at any point between Cincinnati and Toledo. Any desiring to avail themselves of this arrangement, may notify me, and I will procure their tickets.

Arrangements have also been made, we understand, by the Executive committee, with private houses to entertain those who do not wish to stop at the hotels. J. TAFT.

### The Dental Register

OF THE WEST.

*Issued Monthly, at $3 a Year in Advance.*

DEVOTED TO THE INTERESTS OF THE DENTAL PROFESSION.

EDITED BY J. TAFT AND G. WATT.

The volume commences in January and closes in December.

The Register being issued monthly is always fresh, therefore indispensable to the practitioner who desires to keep posted up with the new inventions and improvements which are daily developed. Neither expense nor effort will be spared to give value and variety to its contents. Articles will be illustrated with well executed engravings whenever they will add to the interest or assist in explanation.

Its extensive circulation and frequency of issue makes it one of the BEST DENTAL ADVERTISING MEDIUMS in the United States.

RATES OF ADVERTISING.

One page, 1 year, $50. Half page, 1 year, $30. Quarter page, or card, 1 year $20.

All advertising bills payable in advance, or at furthest after the issue of the first number containing the advertisement. All transient advertisements to be paid for in advance.

☞ CONTRIBUTIONS SOLICITED.

Address, J. TAFT, or "DENTAL REGISTER," Cincinnati Ohio.

Business Communications to

J. TAFT, Publisher,

No. 238 Fourth St., between Plum and Central Avenue

